# Decoding ‘Maximum Entropy’ Deconvolution

**DOI:** 10.3390/e24091238

**Published:** 2022-09-02

**Authors:** Long V. Le, Tae Jung Kim, Young Dong Kim, David E. Aspnes

**Affiliations:** 1 Institute of Materials Science, Vietnam Academy of Science and Technology, Hanoi 100000, Vietnam; 2 Department of Physics, Kyung Hee University, Seoul 02447, Korea; 3 Department of Physics, North Carolina State University, Raleigh, NC 27695-8202, USA

**Keywords:** maximum entropy, deconvolution, spectral analysis

## Abstract

For over five decades, the mathematical procedure termed “maximum entropy” (M-E) has been used to deconvolve structure in spectra, optical and otherwise, although quantitative measures of performance remain unknown. Here, we examine this procedure analytically for the lowest two orders for a Lorentzian feature, obtaining expressions for the amount of sharpening and identifying how spurious structures appear. Illustrative examples are provided. These results enhance the utility of this widely used deconvolution approach to spectral analysis.

## 1. Introduction

In a landmark thesis, Burg developed a formalism based on a maximum-entropy calculation that provided a method of deconvolving spectra, i.e., sharpening dominant features and detecting others too weak to be seen in the data [1]. Despite known limitations, which include unexplained shifts in energies and the appearance of spurious structures, this procedure has been used extensively for over five decades in fields as varied as geophysics [2], astronomy [3,4], X-ray photoelectron spectroscopy [5,6,7,8,9,10], and Raman spectroscopy [11,12,13,14,15,16], among others [17,18,19].

While numerical studies have outlined the general characteristics of the formalism [1,20,21,22], many details are not understood. For example, quantitative predictions of the degree of sharpening do not exist, even for special cases. In this work, we approach this lack of understanding by solving the first- and second-order Burg equations analytically for a Lorentzian feature whose Fourier coefficients are those ($e^{-|n|\Gamma }$) of the continuum. This analysis parallels our previous work on noise reduction using the corrected maximum-entropy (CME) procedure [23], where a similar calculation was found to yield an exact analytic result. The results obtained here are also found to be exact, and are illustrated with representative applications. Surprisingly, the degree of sharpening depends on the ratio of the width of the feature to the width of the spectral segment being analyzed. These results enhance the utility of this widely used deconvolution approach to spectral analysis.

## 2. Theory

The path through the origin, development, and reduction to practice of Burg’s deconvolution theory began with the mid-century goal of extracting weak harmonic signals in stationary time series, where the signals are buried in Gaussian noise. The procedure started with autocorrelation, an operation incompatible with spectroscopy. Applications evolved through multiple steps, including forward prediction [24]. We do not attempt to trace the path here. We consider only the result, cast in a form suitable for the present application. This is Andersen’s approach [22], later extended to complex coefficients by Kesler and Hayken [25].

In spectroscopy, the procedure is based on the Fourier coefficients of the spectrum being analyzed. To define these procedures, let $P_{o}(\theta_{j})=p_{j}$ be a positive-definite spectrum consisting of (2N+1) real values $p_{j}$, −N≤j≤N, projected onto the range −π≤θ≤π according to

(1) $$\theta_{j}=\frac{2\pi }{2N+1}j.$$

The restriction to an odd number (2*N* + 1) of data is required by the maximum-entropy derivation, and is also mathematically convenient. Next, let $R_{n}$, −N≤n≤N be the set of digital Fourier coefficients associated with the $p_{j}$, determined as

(2) $$R_{n}=\frac{1}{2N+1}\sum_{j=-N}^{N}p_{j}e^{-in\theta_{j}}.$$

Because the $p_{j}$ are real, $R_{n}^{*}=R_{-n}$. Then, the CME representation $P_{M}(\theta )$ of $P_{o}(\theta )$ of order *M* is given by [23]

(3) $$P_{M}(\theta )=\frac{1}{{\left|\sum_{n=0}^{M}a_{Mn}^{*}e^{-in\theta }\right|}^{2}},$$

where the $a_{Mn}$ are solutions of

(4) $$\left(\begin{array}{cccc} R_{0} & R_{1} & \ldots & R_{M}\\ R_{-1}^{} & R_{0} & \ldots & R_{M-1}\\ \ldots & \ldots & \ldots & \ldots \\ R_{-M}^{} & R_{-M+1}^{} & \ldots & R_{0} \end{array}\right)\left(\begin{array}{c} a_{M0}\\ a_{M1}^{*}\\ \ldots \\ a_{MM}^{*} \end{array}\right)=\left(\begin{array}{c} 1/a_{M0}\\ 0\\ \ldots \\ 0 \end{array}\right).$$

The Toeplitz (diagonal-constant) form originated with the need to perform autocorrelation in time-sequence applications, and is retained for spectroscopy because maximum-entropy theory requires it. The theory is also best suited to Lorentzian features, because as shown below, Equation (3) reduces to Lorentzian form under small-term conditions.

The solution of these equations is readily obtained either by Levinson recursion [26,27] or by inverting the Toeplitz matrix. The resulting uppermost equation is solved for $a_{M0}$, after which the remaining coefficients are calculated. When the resulting spectrum $P_{M}(\theta )$ of Equation (3) is Fourier analyzed, the original coefficients $R_{0},\;\;R_{1},\;\ldots R_{M}$ are found to be recovered exactly, while those for n>M continue the established trend. As previously shown in [23], it follows that this approach possesses all the advantages of the brick-wall filter with none of the disadvantages, yielding the most accurate noise-free representation of spectral data with no apodization (filter-cutoff) errors.

Burg/Andersen (B/A) deconvolution [22,25] is entirely different, sharing only the starting Toeplitz matrix and the final pseudo-Lorentzian form, Equation (3). Here, the objective is to obtain $a_{Mn}$ such that the denominator of Equation (3) approaches zero as closely as possible at the locations $\theta_{j}$ of the *j* features in the spectrum. The result is a sharpened or “whitened” version of the original. The calculation is a Levinson-like recursion, generating a spectrum P(θ) that when Fourier-analyzed, the original $R_{n}$ are found to be replaced with new values having a smaller decay coefficient, as described below.

The specific B/A procedure is as follows. Assume that the elements $a_{M\mu }$ of the solution vector of the *M*th-order Toeplitz-matrix equation are known, but with the vector normalized to $a_{M0}=1$. The term $1/a_{M0}$ in the right side vector of Equation (4) is replaced by a “power” $P_{M}$ that is also determined from the *M*th-order solution. (The terminology “power” is relevant for stationary time series but has no meaning for spectroscopy). Next, increase the size of $\underset{˜}{R}$ by adding 1 row and 1 column, so M→M+1. Now evaluate

(5) $$b_{Mn}=\sum_{\kappa =0}^{M}a_{M\kappa }^{*}R_{n+\kappa };$$

(6) $$b_{Mn}^{\prime }=\sum_{\kappa =0}^{M}a_{M\kappa }R_{n+M+1-\kappa }.$$

From a stationary-time-sequence perspective, Equation (5) is a filter running in the forward direction, i.e., predicting the output $b_{Mn}$ for increasing κ, whereas Equation (6) is the same filter operating in the reverse direction, predicting $b_{Mn}^{\prime }$ for decreasing κ. Next, evaluate

(7) $$C_{M+1}^{}=\frac{2\sum_{n=0}^{M}b_{M,n}^{*}b_{M,n}^{\prime }}{\sum_{n=0}^{M}\left(|b_{M,n}^{}{|}^{2}+|b_{M,n}^{\prime }{|}^{2}\right)}$$

The (M+1)-order solutions $a_{M+1,n}$, $P_{M+1}$ are then given by

(8) $$a_{M+1,0}=1;$$

(9) $$a_{M+1,n}=a_{M,n}+C_{M+1}a_{M,M+1-n}^{*},\;n=1,\;\;2,\;\;\ldots ,\;M;$$

(10) $$a_{M+1,M+1}=C_{M+1};$$

(11) $$P_{M+1}=P_{M}\left(1-|C_{M+1}{|}^{2}\right).$$

The recursion calculation is initiated with the starting values

(12) $$P_{0}=\sum_{n=0}^{N}|R_{n}{|}^{2}$$

and $a_{00}=a_{10}=1$. The starting value of $a_{11}^{}$ is determined by minimizing

(13) $$\pi_{1}=\frac{1}{2}\sum_{n=0}^{N}\left[{\left|R_{n+1}+a_{11}R_{n}\right|}^{2}+{\left|R_{n}+a_{11}^{*}R_{n+1}\right|}^{2}\right]$$

with respect to $a_{11}^{}$. The results are

(14) $$a_{11}=C_{1}=-\frac{2\sum_{n=0}^{N-1}R_{n}^{*}R_{n+1}}{\sum_{n=0}^{N-1}\left(|R_{n}{|}^{2}+|R_{n+1}{|}^{2}\right)};$$

(15) $$P_{1}=P_{0}(1-|C_{1}{|}^{2}).$$

The calculation then proceeds with Equations (7)–(11). The bidirectional averaging implicit in Equations (5) and (6) minimizes odd-harmonic contributions, leading to deconvolution.

## 3. Analytic Investigation

We now consider analytic solutions, with the objectives of obtaining information about the deconvolution process, properties of the solutions, and useful estimates of the amount of sharpening that can be expected in specific situations. As with our previous work [23], we base our analysis on the Fourier coefficients $e^{-|n|\;\Gamma }$ of the continuum normalized Lorenzian

(16) $$P_{o}(\theta )=\frac{\Gamma }{\pi \left(\theta^{2}+\Gamma^{2}\right)},$$

which for mathematical simplicity we place at the center of the range −π≤θ≤π, defined by Equation (1). The digital Fourier transform $P_{D}(\theta )$ of $e^{-\;|n|\;\Gamma }$ is

(17) $$P_{D}(\theta )=\sum_{n=-N}^{N}e^{-|n|\Gamma +in\theta }$$

(18) $$=\frac{1-e^{-2\Gamma }-2e^{-(N+1)\Gamma }\left(\cos(N+1)\theta -e^{-\Gamma }\cos(N\theta )\right)}{1-2e^{-\Gamma }\cos\theta +e^{-2\Gamma }}.$$

(19) $$≃\frac{1-e^{-2\Gamma }}{1-2e^{-\Gamma }\cos\theta +e^{-2\Gamma }}$$

(20) $$=\frac{\sinh\Gamma }{\cosh\Gamma -\cos\theta }.$$

In Equation (19), it is assumed that (N+1)Γ is large enough that the apodization (“ringing”) term in Equation (18) can be discarded. The M=1 solution $P_{1}(\theta )$ of Equations (3) and (4) is identical to Equation (19), except that it is exact. As shown in [23], with $R_{n}=e^{-|n|\Gamma }$, all higher-order terms vanish. For small Γ and θ, Equation (20) reduces to

(21) $$P_{D}(\theta )\to \frac{2\Gamma }{\Gamma^{2}+\theta^{2}},$$

which differs from Equation (16) only by the (1/2π) normalizing factor of the continuum Fourier transform. Thus, the broadening is the square root of the closest approach of the denominator to zero. The full-width-half-maximum (FWHM) is 2Γ.

We now consider the equivalent B/A *M* = 1 solution using Equations (3) and (14), providing detail as necessary. Substituting $R_{n}=e^{-n\Gamma }$ in Equation (14) yields

(22) $$a_{11}=\frac{2\sum_{n=1}^{N-1}e^{-n\Gamma }e^{-(n+1)\Gamma }}{\sum_{n=1}^{N-1}\left(e^{-2n\Gamma }+e^{-2(n+1)\Gamma }\right)}$$

(23) $$=\frac{2e^{-\Gamma }\sum_{n=1}^{N-1}e^{-2n\Gamma }}{\sum_{n=1}^{N-1}e^{-2n\Gamma }+e^{-2\Gamma }\sum_{n=1}^{N-1}e^{-2n\Gamma }}$$

(24) $$=\frac{2e^{-\Gamma }}{1+e^{-2\Gamma }},$$

(25) $$=\frac{1}{\cosh\Gamma },$$

where Equation (24) follows from Equation (23) by canceling the common sums, and Equation (25) from Equation (24) by multiplying the numerator and denominator by $e^{\Gamma }$. As a result of the special property of $R_{n}=e^{-|n|\Gamma }$, the sums in Equation (23) cancel regardless of the initial and final values of *n*, so the resulting Equation (25) is independent of *N*.

The remaining mathematics is more efficiently carried out in two parts, evaluating first the power prefactor $P_{1}$ and next the denominator $D_{1}$. For the prefactor, we find

(26) $$P_{1}=\left(1-a_{11}^{2}\right)\left(\sum_{n=1}^{N}e^{-2n\Gamma }\right),$$

(27) $$≃\frac{e^{\Gamma }\sinh\Gamma }{2\cosh^{2}\Gamma },$$

assuming that *N* is large enough that the apodization term in the numerator can be ignored. Here, the sum depends on the starting value of *n*. We can remove this ambiguity by noting that the digital transform Equation (2) uses all terms −N≤n≤N, whereas Equation (26) is single-ended. Consistency is achieved by taking the average of sums starting at n=0 and n=1, in which case each term is considered once and only once. The result is

(28) $$P_{1}=\frac{1}{2}\left(e^{\Gamma }+e^{-\Gamma }\right)\frac{\sinh\Gamma }{2\cosh^{2}\Gamma }$$

(29) $$=\frac{1}{2}\tanh\Gamma .$$

The contribution of the denominator is

(30) $$D_{1}=\frac{1}{{\left|1-\frac{1}{\cosh\Gamma }e^{-i\theta }\right|}^{2}}$$

(31) $$=\frac{\cosh^{2}\Gamma }{\cosh^{2}\Gamma -2\cosh\Gamma \cos\theta +1}.$$

Combining the two expressions yields the M=1 lineshape $P_{M}(\theta )$, i.e., the M=1 deconvolved version of $P_{o}(\theta_{j})$:

(32) $$P_{1}(\theta )=\frac{\sinh2\Gamma }{4\left(\cosh^{2}\Gamma -2\cosh\Gamma \cos\theta +1\right)}.$$

The structural similarity between Equations (19) and (32) is evident.

This equation is best understood by repeating the small-term expansion leading to Equation (21) in the CME case. Performing the series expansions of sinhΓ, coshΓ, and cosθ for small Γ and θ, we find

(33) $$P_{1}(\theta )≃\frac{2\Gamma -\frac{4}{3}\Gamma^{3}}{4\left(\frac{\Gamma^{4}}{4}+\frac{\Gamma^{2}\theta^{2}}{2}+\theta^{4}\right)}$$

(34) $$=\frac{\frac{\Gamma }{2}-\frac{\Gamma^{3}}{3}}{{\left(\frac{\Gamma^{2}}{2}\right)}^{2}+\theta^{2}\left(1+\frac{\Gamma^{2}}{2}\right)-\frac{\theta^{4}}{12}}.$$

Ignoring the small $\theta^{4}$ term in the denominator, the lineshape is seen to be Lorentzian, with a FWHM of

(35) $${\mathrm{FWHM}}_{B/A}=\frac{\Gamma^{2}}{\sqrt{1+\Gamma^{2}/2}}.$$

This can be compared to the FWHM of 2Γ for the original Lorentzian. The relative narrowing is more relevant, so we divide Equation (35) by 2Γ, which yields

(36) $$\frac{{\mathrm{FWHM}}_{B/A}}{{\mathrm{FWHM}}_{original}}=\frac{\Gamma }{\sqrt{4+2\Gamma^{2}}}$$

(37) $$\sim \frac{\Gamma }{2},$$

where in Equation (37) it is assumed that the second term in the radical can be ignored relative to the first. Since the dimensionless reference for Γ is the Fourier period 2π, we find the surprising result that the relative sharpening is also a function of the width of the structure relative to the width of the spectral segment being analyzed. For structures with values of Γ that are small compared to the intrinsic reference scale of 2π, the sharpening can be significant.

While the M=1 B/A FWHM is obviously less than the original for Γ<1, a short calculation shows that it is always the case if

(38) $$4+\Gamma^{2}>0.$$

Because this inequality is satisfied for any Γ, the B/A process for M=1 always results in a narrower Lorentzian line.

Because the width and peak values of the Lorentzian are related, narrowing is equivalent to moving the singularity in the denominator closer to zero. At θ=0, we find

(39) $$P_{1}(0)=\frac{\sinh\Gamma }{4{\left(\cosh\Gamma -1\right)}^{2}}≃\frac{1}{4\Gamma^{3}},$$

compared to 1/π Γ for the original normalized Lorentzian. The pole has thus moved 2 inverse powers of Γ closer to zero, consistent with the decreased width.

At this point we note the importance of averaging: had normalization been performed using only the first term in the denominator of Equation (23), the result would be $a_{11}=e^{-\Gamma }$, yielding the CME result Equation (19). Had Equation (23) been normalized by the second term, the result would be $a_{11}=e^{\Gamma }>1$, violating a fundamental constraint of the theory. In essence, the B/A approach works because of averaging.

It is straightforward to take the calculation one step further, determining the result for M=2. Using the same strategy, we find

(40) $$a_{21}=\frac{2\cosh\Gamma }{\cosh2\Gamma };$$

(41) $$a_{22}=-\frac{1}{\cosh2\Gamma };$$

(42) $$P_{2}=P_{1}(1-a_{22}^{2})$$

(43) $$=\frac{\sinh\Gamma \sinh^{2}2\Gamma }{2\cosh\Gamma \cosh^{2}2\Gamma };$$

(44) $$D_{2}=\frac{\cosh^{2}2\Gamma }{{\left|\cosh2\Gamma -2\cosh\Gamma e^{-i\theta }+e^{-i2\theta }\right|}^{2}};$$

whence

(45) $$P_{2}(\theta )=\frac{\sinh\Gamma \sinh^{2}2\Gamma }{2\cosh\Gamma {\left|\cosh2\Gamma -2\cosh\Gamma e^{-i\theta }+e^{-i2\theta }\right|}^{2}}.$$

This expression is significantly more complicated than that for $P_{1}(\theta )$, although its properties are easily determined. We consider first the extrema, which can be found by setting the derivative of the denominator with respect to θ equal to zero. The calculation yields

(46) $$0=\left(2i\cosh\Gamma e^{-i\theta }-2ie^{-2i\theta }\right)\left(\cosh2\Gamma -2\cosh\Gamma e^{i\theta }+e^{2i\theta }\right)\;\;+\;\;c.c.$$

(47) $$=2\sin\theta \left(\cosh\Gamma \cosh2\Gamma -2\cosh2\Gamma \cos\theta +\cosh\Gamma \right).$$

The factored term sinθ shows that one extremum occurs at θ=0, as expected by symmetry. However, Equation (47) also has second and third extrema at locations given by

(48) $$\cos\theta =\frac{1}{2}\left(1+\frac{\cosh\Gamma }{\cosh2\Gamma }\right).$$

Because cosh2Γ>coshΓ for Γ>0, it follows that the right side of Equation (48) is less than 1 under all conditions, hence by symmetry this solution is valid for any Γ. Thus for M=2, the reconstruction of the single Lorentzian is split into two peaks.

We next consider the small-term expansion for M=2. Expanding Equation (47) to fourth order in Γ and θ, we find

(49) $$P_{2}(\theta )≃\frac{4\Gamma^{3}}{{\left(\theta^{2}-\Gamma^{2}\right)}^{2}}.$$

The denominator is the square of a parabola with singularities at θ=±Γ. This is consistent with the above analysis: the single peak of the original Lorentzian has split into two, with each new singularity located a distance Γ from the symmetry axis. The infinities at θ=±Γ in Equation (45) are eliminated in a 6th-order expansion of the denominator, which yields the more accurate small-term result

(50) $$P_{2}(\theta )≃\frac{4\Gamma^{3}}{{\left(\theta^{2}-\Gamma \prime^{2}\right)}^{2}+4\Gamma^{6}},$$

where

(51) $$\Gamma \prime =\Gamma \left(1-\sqrt{7\Gamma^{2}/3}\right).$$

Because $P_{2}(\Gamma \prime )=\frac{1}{\Gamma^{3}}>P_{2}(0)=\frac{4\Gamma^{3}}{\Gamma \prime^{4}}$, the location θ=0 is a local minimum.

Thus, for M=2, the singularities are moved even closer to zero, although the price paid is that two peaks are present instead of one. While it is possible to obtain an expression yielding the FWHM of each peak, the splitting has made the concept of sharpening irrelevant. We note that in going from M=1 to M=2, the peak went from being the reciprocal of a fourth-order quantity to the reciprocal to a sixth-order quantity.

Next, we note that small-term expansions may be expected to have limited utility. As an alternative, replacing $e^{i\theta }\to z$ and setting the denominator equal to zero yields

(52) $$z_{\pm }=\cosh\Gamma \pm \sqrt{\cosh^{2}\Gamma -\cosh2\Gamma }$$

(53) =coshΓ±isinhΓ.

Again, we see that the result contains two peaks, which are now separated by Δθ=sinhΓ.

The above calculations are too crude to have significant quantitative value, but they illustrate a basic mechanism that we expect to be valid in other situations: when too many peaks (too large a value of *M*) are requested, the existing peaks are not only shifted, but extra features appear. We expect this to apply to orders of *M* beyond 2 as well.

## 4. Discussion

**Figure 1:**
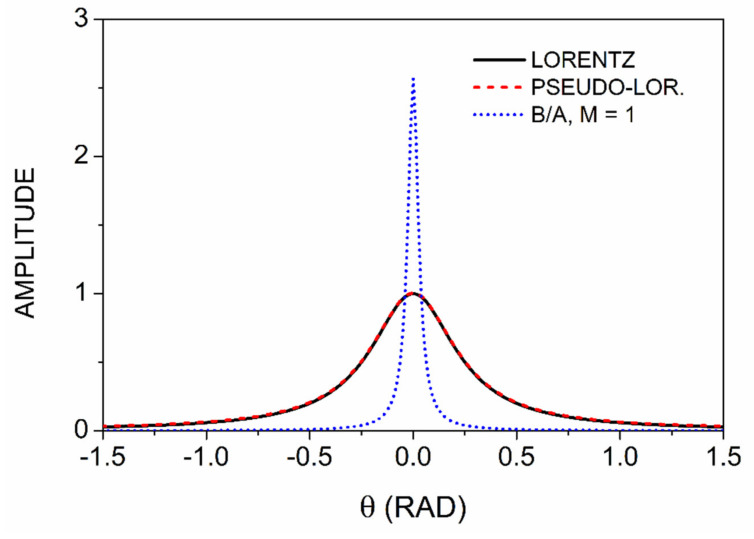
Comparison of Lorentzian, pseudo-Lorentzian, and B/A lineshapes.

To better visualize the nature of the solutions, we turn to numerical methods. Figure 1 compares the normalized deconvolved spectrum for M=1, calculated from Equation (32), to the original Lorentzian and the associated pseudo-Lorentzian calculated from Equations (16) and (19), respectively. The curves are shown on the expanded range −1.5≤θ≤1.5 to better display differences. On this scale, the Lorentzian and the pseudo-Lorentzian are essentially identical, but the deconvolved result is larger than the others by a factor of about 2½. From Equation (35) the linewidth is reduced by a factor of 8.1, in agreement with Figure 1. It can be noted from Equation (35) that the extreme sharpening seen here is a direct consequence of the relatively small value Γ=0.25 relative to the ± π range of *θ*. Had we chosen a larger value of Γ, the relative amount of sharpening would have been less.

**Figure 2:**
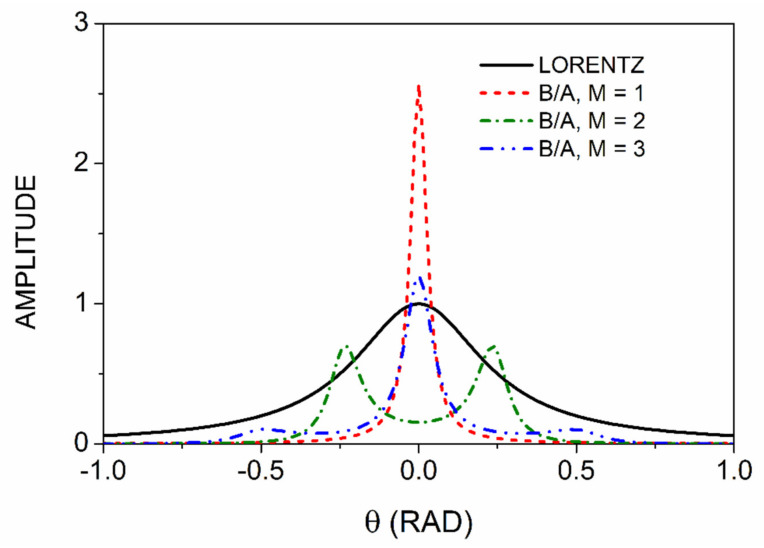
B/A processing of the single Lorentzian (bottom spectrum) for *M* = 1, 2, and 3. All lineshapes are to scale.

As noted above, when *M* exceeds the number of singularities, the system generates extra poles. Figure 2 illustrates this in detail. Here, we show the results of the B/A procedure for M=1,  2, and 3, comparing the results to each other and to the original Lorentzian at the bottom. The enhancement of the amplitude of the M=1 spectrum with respect to the original Lorentzian is clear. Another striking feature is that the curve for M=2 has two peaks instead of one, with the peaks located approximately at θ=±Γ,as shown in the previous section. The curve for M=3 recovers the single dominant peak, but its amplitude is reduced by about a factor of 2. In addition, spurious satellites appear at θ=±2Γ. Thus, if *M* exceeds the number of legitimate critical points in the spectrum, the result is not only extra features, but also a significantly reduced peak height when a feature coincides with a structure of the spectrum.

**Figure 3:**
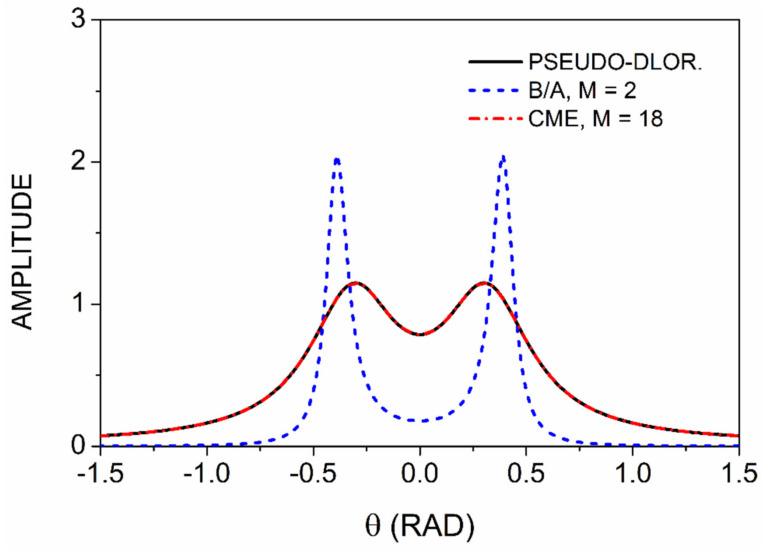
Result of B/A processing of a double-peaked model spectrum. Details are given in the text.

Because spectra usually contain more than one feature, the question arises as to how much of the simple single-line theory is transferrable to spectra containing multiple structures. Figure 3 shows a model spectrum consisting of a pair of lines with Γ=0.25, symmetrically located about the origin with a separation of Δθ=±π/10=±0.314. This is sufficient for the peaks to remain distinct while retaining some overlap. It is seen that the M=2 lineshapes are much sharper than the original, so the B/A procedure is effective. However, an examination of the numerical values shows that the broadening is about 0.042, leading to a ratio of 0.17 compared to the predicted value of 0.123. The difference is about 30%. In addition, the singularities are found to lie at about ±0.38 from the symmetry axis instead of ±0.314, a difference of 17%. Although no measures of broadening have appeared before, errors in the locations of singularities are well-known. We shall discuss this in more detail elsewhere.

**Figure 4:**
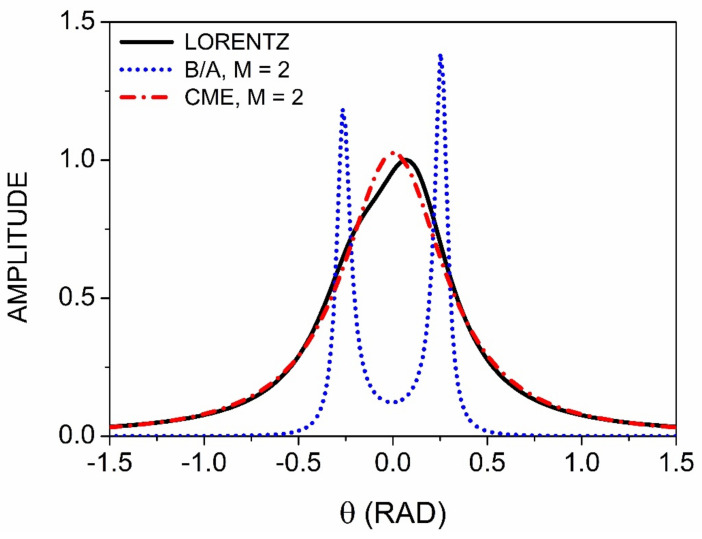
Results of B/A and CME processing of a double-peaked model spectrum. Details are given in the text.

Figure 4 compares the capabilities of the CME and B/A approaches to deal with singularities separated essentially by the broadening parameter. The synthetic spectrum consists of two poles, the first at θ=−0.20 with an amplitude of 0.5, and the second at $\theta_{2}=+0.10$ with an amplitude of 1.0. The broadening parameter Γ=0.25 is the same in both cases, and both calculations were performed to the order M=2. The CME is functioning as it should, i.e., generating a replica of the data with white-noise coefficients replaced with most-probable values. The B/A response is qualitatively different, with the sharpening evident and the two singularities clearly identified. However, the B/A procedure places these at −0.267 and +0.258, which is only qualitatively correct. The CME performs better, but only at higher ***M***. For M=20, two poles are also identified, located at −0.233 and +0.110, in better agreement with the correct values.

## 5. Conclusions

In this work, we investigate the B/A procedure analytically for a single Lorentzian line and B/A orders M=1 and 2. We take advantage of the form $e^{-|n|\;\Gamma }$ of the continuum Fourier coefficients of the Lorentz lineshape to obtain exact analytic expressions. In both cases, the Lorentz structure is deconvolved, with the amount of sharpening greater for M=2 than M=1. The M=1 case yields a simple analytic expression that can be used to estimate the amount of deconvolution that can be expected in given situations. Surprisingly, the relative amount of deconvolution is not intrinsic to the structure but depends on the ratio of the width of the original structure to the width of the spectral segment being analyzed, not just on the width of the structure itself.

The origin of spurious peaks is demonstrated to be the result of selecting an order that does not match the number of singularities in the structure, with degradation seen first as a splitting of a single peak, followed at higher orders with the appearance of satellite structures and reductions in the enhancement of the main peak. In applications to a model spectrum and data, we find that when two peaks are present, B/A processing for order 2 highlights the structures, in contrast to the CME, although for large *M* the CME returns more accurate values of the singularities.

## Data Availability

Not applicable.
